# Enhanced estimation strategy for determining the location of tracheoesophageal fistula in a preterm, low-birth-weight infant with congenital esophageal atresia type C and duodenal atresia: a case report

**DOI:** 10.1186/s40981-024-00730-3

**Published:** 2024-07-30

**Authors:** Seirin Yamazaki, Yusuke Miyazaki, Yoshie Taniguchi, Shoichi Uezono

**Affiliations:** Department of Anesthesiology, The Jikei University School of Medicine, Tokyo, Japan

**Keywords:** Tracheoesophageal fistula, Esophageal atresia, Preterm, Low-birth-weight infant

## Abstract

**Background:**

In esophageal atresia type C, identifying the tracheoesophageal fistula (TEF) location is crucial for airway management. However, a thin bronchoscope may not always be available.

**Case presentation:**

We report on a low-birth-weight neonate with esophageal atresia type C who required immediate gastrostomy after birth. With no suitable thin bronchoscope available, alternative methods were utilized to estimate the TEF location post-gastrostomy. Submerging the gastrostomy tube tip in water and applying positive pressure ventilation via a tracheal tube allowed for observation of air bubbles emerging from the gastrostomy tube. As the tracheal tube was advanced, the cessation of bubbles indicated that the TEF was sealed by the tracheal tube. The location of the tracheal tube tip, confirmed by chest radiographs, was consistent with the TEF location identified during corrective surgery for TEF.

**Conclusions:**

This innovative technique facilitated successful estimation of the TEF location without bronchoscopy, demonstrating its efficacy in resource-limited settings.

## Background

Esophageal atresia is one of the most rarely diagnosed congenital anomalies [1–3]. Specifically, patients presenting with esophageal atresia alongside a concurrent tracheoesophageal fistula (TEF) are at increased risk for severe complications, such as gastric rupture or cardiac arrest as a result of gastric distention and compromised ventilation induced by positive pressure ventilation during anesthesia [4, 5]. Typically, diagnostic imaging such as computed tomography scan or fiber-optic bronchoscopy is used to determine the location of a TEF [6–8]. To mitigate gastric insufflation, a TEF can be temporarily sealed with a tracheal tube or occluded with a Fogarty catheter [9–11]. Consequently, general anesthesia would become more straightforward to manage when the location of a TEF is established.

We report a case in which comprehensive evaluations, including computed tomography and fiber-optic bronchoscopy, were unfeasible due to the urgency of surgical intervention in a patient diagnosed with esophageal atresia type C and concomitant duodenal atresia. Estimating a TEF location was achievable through monitoring gas efflux from a gastrostomy tube post-gastrostomy, correlated with positive pressure ventilation via a tracheal tube. This case report follows the Anaesthesia Case Report checklist [12].

## Case presentation

The patient was a 0-day-old male neonate born at 34 weeks and 4 days of gestation by emergency caesarean section due to chorioamnionitis. He weighed 2075 g at birth, measured 48.5 cm in length, and received Apgar scores of 8 at 1 min and 8 at 5 min, with a cord blood pH of 7.352. The patient was admitted to the neonatal intensive care unit (NICU) because of premature birth, low birth weight, and duodenal atresia confirmed by prenatal ultrasound examination. A preoperative chest radiograph revealed a double bubble sign and a gasless abdomen. Additionally, a gastric tube was noted to be coiled, and an upper gastrointestinal contrast imaging showed the esophagus terminating in a blind end. These findings indicated the presence of esophageal atresia type C and duodenal atresia. An urgent pediatric surgical consultation resulted in an emergency gastrostomy for gastric decompression. Consequently, preoperative evaluation for the location of a TEF was unattainable.

Following standard monitoring with pulse oximetry, electrocardiography, blood pressure, and body temperature measurements, general anesthesia was induced by rapid induction with midazolam 0.5 mg, fentanyl 5 µg, and rocuronium 3 mg. Mask ventilation was performed with low airway pressure and low tidal volume to minimize gastric insufflation via the TEF. Tracheal intubation was successfully performed in a single attempt using an Airway Scope (Pentax, Tokyo, Japan). A 2.5-mm uncuffed tracheal tube was intubated and secured at a depth of 8 cm after ensuring an adequate air leak. Given the absence of a readily available fiber-optic bronchoscope with an outer diameter less than 2.5 mm, it was impossible to ascertain the TEF location. Intraoperative ventilatory settings were adjusted to pressure-controlled ventilation, with a peak airway pressure of 15 cmH_2_O and positive end-expiratory pressure of 3 cmH_2_O. Anesthesia was maintained with sevoflurane, fentanyl, and rocuronium. Thereafter, the gastrostomy was completed without complications such as inadequate ventilation or notable gastric distention. The surgical duration was 46 min, and the anesthesia duration was 2 h and 10 min.

**Fig. 1:**
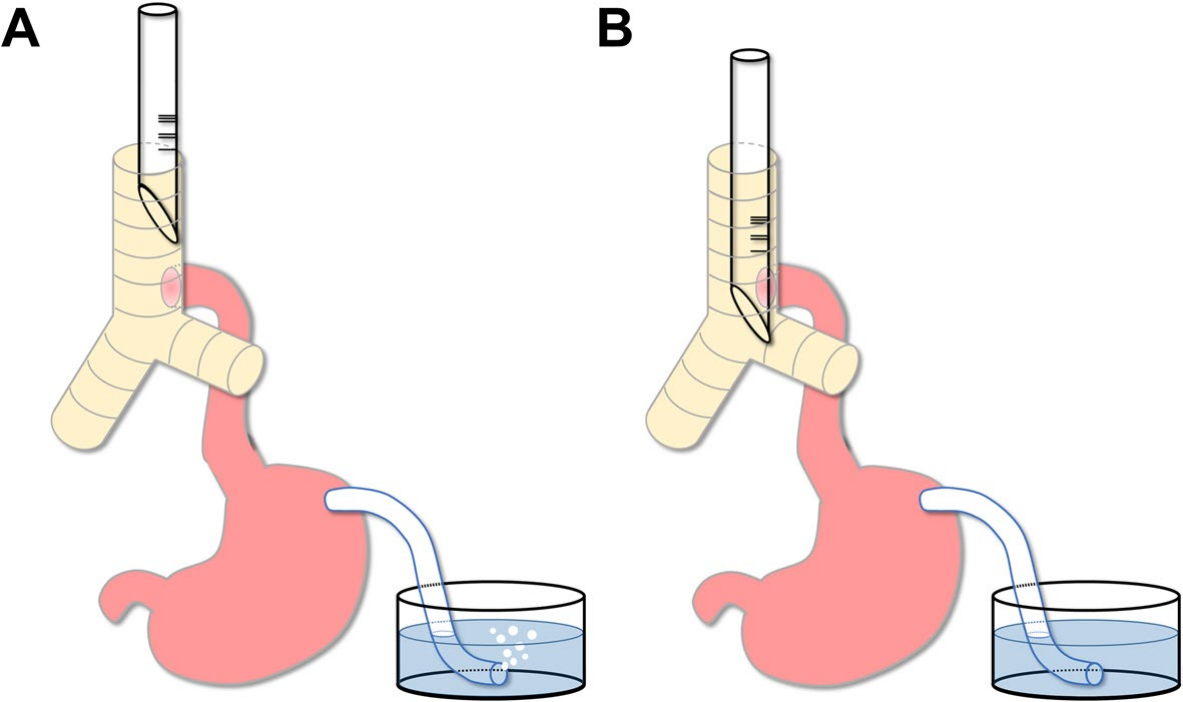
Diagrams illustrating methods for estimating the location of a tracheoesophageal fistula. **A** This diagram illustrates a scenario in which a tracheoesophageal fistula (TEF) is positioned below the tip of a tracheal tube. In this configuration, air bubbles emerging from a gastrostomy tube indicate the presence of the TEF beneath the tip of the tracheal tube. **B** This diagram depicts the situation where the tip of the tracheal tube surpasses the location of the TEF. The absence of air bubbles from the gastrostomy tube signals that the tip of the tracheal tube has moved beyond the TEF

After performing the gastrostomy in the operating room, we employed a method to estimate the location of the TEF. Initially, the distal end of a gastrostomy tube was submerged in water. Subsequently, we assessed whether advancement of the tracheal tube would halt the release of air bubbles from the gastrostomy tube during positive pressure ventilation. Specifically, when the TEF is positioned below the tip of the tracheal tube, air bubbles would be observed emerging from the gastrostomy tube (Fig. 1A). On the other hand, when the tip of the tracheal tube surpasses the TEF, air bubbles would cease to emerge from the gastrostomy tube (Fig. 1B).

In the present case, the emergence of air bubbles ceased when the tracheal tube depth reached 10.5 cm. Subsequent chest radiography confirmed that the tip of the tracheal tube was positioned just above the tracheal bifurcation, thereby suggesting the location of the TEF. The patient was then returned to the NICU without undergoing extubation and experienced no complications.

On postoperative day 2, the patient underwent corrective surgery for TEF, esophageal anastomosis, and duodenal anastomosis, as well as the correction of intestinal malrotation, under general anesthesia. Intraoperative findings corroborated that the TEF was located at the level of the confluence of the azygos vein and the superior vena cava, just above the tracheal bifurcation.

## Discussion

In the present case, the patient presented with esophageal atresia type C concurrently with duodenal atresia. In such case, the gas delivered into the stomach via a TEF lacks an outlet due to the simultaneous obstruction of the duodenum and the esophagus. Consequently, the risk of gastric distension and rupture increases significantly [13–15]. This necessitates a heightened level of vigilance in airway and respiratory management. Therefore, considering the patient was scheduled for ongoing ventilatory management in the NICU post-gastrostomy and was also slated for corrective surgery for TEF, we believed it was beneficial to estimate the TEF location after the gastrostomy through the approaches presented.

The location of the TEF was verified at the level of the junction between the superior vena cava and the azygos vein during the corrective surgery for TEF, indicating that our estimation methods were efficacious [16]. A prior study has indicated that in approximately 88% of esophageal atresia cases, a TEF was located above the tracheal bifurcation [17]. On the other hand, in 12% of cases, a TEF was situated below the tracheal bifurcation, exclusively in the right main bronchus, with no instances reported in the left main bronchus [17]. Given the high probability of a TEF being present in the right main bronchus when situated below the tracheal bifurcation, it becomes possible to approximate the TEF location by advancing a tracheal tube.

In addition to the methods described in this report, both fiber-optic and rigid bronchoscopes can be applicable [18, 19]. However, the use of a fiber-optic bronchoscope is precluded when the inner diameter of the tracheal tube is narrow, as demonstrated in this case. Furthermore, a rigid bronchoscopy, ideally conducted under general anesthesia with preserved spontaneous respiration, is not feasible in facilities lacking a rigid pediatric bronchoscope. While our method requires a pre-established gastrostomy, it offers a practical alternative for locating a TEF when equipment for narrow-diameter fiber-optic or rigid bronchoscopy is unavailable.

In the current case, a 2.5-mm inner diameter tracheal tube was utilized. Consequently, locating the TEF via a fiber-optic bronchoscope was not feasible at our facility, which lacked a readily available fiber-optic bronchoscope with an outer diameter of 2.2 mm. Although we could have used a 2.5-mm fiber-optic bronchoscope by switching to a 3.0-mm inner diameter tracheal tube, we chose not to switch, as the 2.5-mm tube provided adequate positive pressure ventilation with a manageable air leak.

We recognize several limitations of the methods. Firstly, the practice of blindly advancing a tracheal tube may precipitate complications such as gastric distention and rupture resulting from positive pressure ventilation. However, as evidenced in the present case, the gastrostomy tube facilitated the evacuation of intragastric gas, enabling the prevention of severe complications. Secondly, if the tracheal tube inadvertently strays into a TEF, air bubbles may continuously emanate from the gastrostomy tube. Under such circumstances, the misplacement would be detected through a reduction in the end-tidal carbon dioxide concentration. Thirdly, if a TEF is in the right main bronchus and the tracheal tube is advanced to the left main bronchus, no air bubbles would emerge from the gastrostomy tube, potentially leading to misidentification of the TEF location. Thus, if air bubbles cease after left main bronchus intubation, intubation of right main bronchus may be needed to locate the TEF.

Based on the foregoing considerations, we advocate for the technique of blindly advancing a tracheal tube and estimating the location of a TEF by observing gas outflow from a gastrostomy tube submerged in water, synchronized with positive pressure ventilation. This estimation strategy would enable the identification of a TEF location without the need for a thin fiber-optic bronchoscope. Although this method should be performed with a high degree of caution by anesthesiologists, this technique would be especially useful in environments where medical resources are limited. Further investigation into this technique is warranted.

## Data Availability

The data used in this report are available from the corresponding author upon reasonable request.

## Abbreviations

NICU: Neonatal intensive care unit
TEF: Tracheoesophageal fistula

## References

[1] Cassina M, Ruol M, Pertile R, Midrio P, Piffer S, Vicenzi V, et al. Prevalence, characteristics, and survival of children with esophageal atresia: a 32-year population-based study including 1,417,724 consecutive newborns. Birth Defects Res A Clin Mol Teratol. 2016;106:542–8. 26931365 10.1002/bdra.23493

[2] Lupo PJ, Isenburg JL, Salemi JL, Mai CT, Liberman RF, Canfield MA, et al. Population-based birth defects data in the United States, 2010–2014: a focus on gastrointestinal defects. Birth Defects Res. 2017;109:1504–14. 29152924 10.1002/bdr2.1145 PMC5915361

[3] van Lennep M, Singendonk MMJ, Dall’Oglio L, Gottrand F, Krishnan U, Terheggen-Lagro SWJ, et al. Oesophageal atresia. Nat Rev Dis Primers. 2019;5:26. 31000707 10.1038/s41572-019-0077-0

[4] Tandon RK, Sharma S, Sinha SK, Rashid KA, Dube R, Kureel SN, et al. Esophageal atresia: factors influencing survival—experience at an Indian tertiary centre. J Indian Assoc Pediatr Surg. 2008;13:2–6. 20177477 10.4103/0971-9261.42564 PMC2810819

[5] Knottenbelt G, Costi D, Stephens P, Beringer R, Davidson A. An audit of anesthetic management and complications of tracheo-esophageal fistula and esophageal atresia repair. Paediatr Anaesth. 2012;22:268–74. 22098314 10.1111/j.1460-9592.2011.03738.x

[6] Atzori P, Iacobelli BD, Bottero S, Spirydakis J, Laviani R, Trucchi A, et al. Preoperative tracheobronchoscopy in newborns with esophageal atresia: does it matter? J Pediatr Surg. 2006;41:1054–7. 16769333 10.1016/j.jpedsurg.2006.01.074

[7] Su P, Huang Y, Wang W, Zhang Z. The value of preoperative CT scan in newborns with type C esophageal atresia. Pediatr Surg Int. 2012;28:677–80. 22491897 10.1007/s00383-012-3082-x

[8] Sharma N, Srinivas M. Laryngotracheobronchoscopy prior to esophageal atresia and tracheoesophageal fistula repair—its use and importance. J Pediatr Surg. 2014;49:367–9. 24528988 10.1016/j.jpedsurg.2013.09.009

[9] Salem MR, Wong AY, Lin YH, Firor HV, Bennett EJ. Prevention of gastric distention during anesthesia for newborns with tracheoesophageal fistulas. Anesthesiology. 1973;38:82–3. 4564847 10.1097/00000542-197301000-00020

[10] Rinkel R, Van Poll D, Sibarani-Ponsen R, Sleeboom C, Bakx R. Bronchoscopy and fogarty balloon insertion of distal tracheo-oesophageal fistula for oesophageal atresia repair with video illustration. Ann Otol Rhinol Laryngol. 2017;126:6–8. 27821414 10.1177/0003489416669951

[11] Edelman B, Selvaraj BJ, Joshi M, Patil U, Yarmush J. Anesthesia practice: review of perioperative management of H-type tracheoesophageal fistula. Anesthesiol Res Pract. 2019;2019:8621801. 31781201 10.1155/2019/8621801 PMC6875187

[12] Shelton CL, Klein AA, Bailey CR, El-Boghdadly K. The Anaesthesia Case Report (ACRE) checklist: a tool to promote high-quality reporting of cases in peri-operative practice. Anaesthesia. 2021;76:1077–81. 33440026 10.1111/anae.15391

[13] Akcora B, Eris O. A newborn with duodenal atresia and a gastric perforation. Afr J Paediatr Surg. 2010;7:33–5. 20098009 10.4103/0189-6725.59359

[14] Nabzdyk CS, Chiu B, Jackson CC, Chwals WJ. Management of patients with combined tracheoesophageal fistula, esophageal atresia, and duodenal atresia. Int J Surg Case Rep. 2014;5:1288–91. 25460495 10.1016/j.ijscr.2013.09.016 PMC4275966

[15] Doval L, Rousseau V, Irtan S. Combined esophageal and duodenal atresia: a review of the literature from 1950 to 2020. Arch Pediatr. 2023;30:420–6. 37328325 10.1016/j.arcped.2023.05.004

[16] Şener U, Tellioğlu AM, Polat YD. A reappraisal of pediatric thoracic surface anatomy. Clin Anat. 2023;36:178–89. 36088577 10.1002/ca.23950

[17] Holzki J. Bronchoscopic findings and treatment in congenital tracheo-oesophageal fistula. Pediatr Anesth. 1992;2:297–303. 10.1111/j.1460-9592.1992.tb00220.x

[18] Barbato A, Magarotto M, Crivellaro M, Novello A Jr, Cracco A, de Blic J, et al. Use of the paediatric bronchoscope, flexible and rigid, in 51 European centres. Eur Respir J. 1997;10:1761–6. 9272916 10.1183/09031936.97.10081761

[19] Singh V, Singhal KK. The tools of the trade—uses of flexible bronchoscopy. Indian J Pediatr. 2015;82:932–7. 26286177 10.1007/s12098-015-1869-1

